# Effect of CYP2C9, VKORC1, and CYP4F2 polymorphisms on warfarin maintenance dose in children aged less than 18 years: a protocol for systematic review and meta-analysis

**DOI:** 10.1186/s13643-016-0280-y

**Published:** 2016-06-23

**Authors:** Masanobu Takeuchi, Tohru Kobayashi, Leonardo R. Brandão, Shinya Ito

**Affiliations:** Division of Clinical Pharmacology and Toxicology, The Hospital for Sick Children, 555 University Ave, Toronto, Ontario M5G 1X8 Canada; Division of Pediatric Hematology/Oncology, The Hospital for Sick Children, 555 University Ave, Toronto, Ontario M5G 1X8 Canada

**Keywords:** Warfarin, CYP2C9, VKORC1, CYP4F2, Polymorphism, Children, Meta-analysis

## Abstract

**Background:**

Despite its shortcomings, warfarin is still the most commonly prescribed anticoagulant to prevent thromboembolism in children. In adults, numerous studies confirmed the robust relationship between warfarin maintenance doses and single nucleotide polymorphisms of cytochrome P450 2C9 (CYP2C9), vitamin K epoxide reductase (VKORC1), and cytochrome P450 4F2 (CYP4F2). However, their effect in children still remains to be determined. The primary objective of the present systematic review and meta-analysis is to assess the effect of genotypes of CYP2C9, VKORC1, and CYP4F2 on warfarin maintenance dose in children.

**Methods/design:**

A comprehensive literature review search using the OVID platform will be conducted by a specialized librarian, without language restrictions (i.e., MEDLINE/EMBASE/Cochrane Central Register of Controlled Trials), and all abstracts will be reviewed by two authors. Data abstraction from each eligible study will be extracted individually by two authors (MT and TK), and disagreements will be resolved through discussion with a third person (SI). Critical appraisal of the included analysis of the primary objective will follow the Newcastle–Ottawa Scale, in addition to the Strengthening the Reporting of Genetic Association study (STREGA) statement, and data reporting will follow the Preferred Reporting Items for Systematic Reviews and Meta-Analyses (PRISMA) statement. For the meta-analysis, the presence vs. absence of each genetic polymorphism will be pursued, respectively, using a random effect model with effect size expressed as a mean difference plus 95 % confidence interval.

**Discussion:**

Our study will provide a comprehensive systematic review and meta-analysis on the potential effects of CYP2C9, VKORC1, or CYP4F2 on the warfarin maintenance dose in children, exploring the feasibility of the development of pharmacogenetic-guided warfarin dosing algorithm for children on oral vitamin K antagonists.

**Systematic review registration:**

The review has been registered with PROSPERO (registration number CRD42015016172).

**Electronic supplementary material:**

The online version of this article (doi:10.1186/s13643-016-0280-y) contains supplementary material, which is available to authorized users.

## Background

The diagnosis of thromboembolism (TE) has been increasing in the world, and a considerable portion of those events includes the pediatric population [1, 2]. Among the anticoagulant agents used for the treatment of TE in children, warfarin is the most commonly prescribed oral anticoagulant for the treatment and prevention, including primary anticoagulant prophylaxis for patient with heart valve implants, single ventricle pathology which underwent palliative surgery (e.g., post-Fontan operation), coronary aneurysm of Kawasaki disease, and dilated cardiomyopathy or for children with thrombotic events on therapeutic anticoagulation [3–5]. However, one of the challenges related to the use of warfarin pertains to its low therapeutic index and the large inter-individual variability in maintenance dose requirement [6]. For practical purposes, an insufficient dose of warfarin would result in failure to prevent thrombosis, while overdosing might cause life-threatening hemorrhages [7]. Although the variation in the initial and maintenance dose is known to depend on body weight, age, and dietary vitamin K intake as well as on environmental factors such as concomitant medications, there is growing evidence for a strong hereditary component [8–10].

Warfarin is a racemic mixture of R and S warfarin. The average elimination half-life of S warfarin is 29 h, and that of R warfarin is 45 h. The overall half-life is between 36 and 42 h. It is rapidly absorbed from the gut, and its peak concentration is reached in 60 to 90 min [11]. Over the last decade, a large body of evidence has accumulated showing that polymorphism in three genes influence warfarin dose. The three genes responsible for its metabolism and efficacy are as follows: cytochrome P450 2C9 (CYP2C9) is responsible for the metabolism of S warfarin, vitamin K epoxide reductase complex subunit 1 (VKORC1) is the molecular target of warfarin, and cytochrome P450 4F2 (CYP4F2) is the primary vitamin K_1_ oxidase in the liver. Based on these findings, several pharmacogenetic approaches to guide optimal warfarin maintenance dose have been developed [12–19]. In 2007, the US Food and Drug Administration modified the warfarin label, stating that CYP2C9 and VKORC1 genotypes may be useful to determine the optimal initial dose of warfarin [20]. The label was updated in 2010 describing recommendations for initial dosing ranges for patients with different combinations of CYP2C9 and VKORC1 genotypes. Subsequently, the Clinical Pharmacogenetics Implementation Consortium published the dosing guideline [21] and also recommended the use of pharmacogenetic test in determining initial warfarin doses in adults.

In children, although several pharmacogenetic studies exploring effects of genotypes on warfarin maintenance doses have been reported [22–30], the effect of genotypes on warfarin remains controversial. Given the variable findings of the genotype–phenotype relationship in warfarin therapy in children, critical analyses of the existing information would be of utmost importance to provide clinicians an as-close-as-possible evidence-based view of the literature, allowing them to critically evaluate the need for this information to be incorporated into the care of children being treated with warfarin. The pursuit of such manuscript containing a systematic review and meta-analysis on this issue has motivated us to plan for this study.

Our primary objective is to evaluate the effects of CYP2C9, VKORC1, and CYP4F2 genotypes on warfarin maintenance dose in children aged less than 18 years of age.

## Methods/design

We will conduct a systematic review and meta-analysis following the principles of the Cochrane Handbook for Systematic Reviews of Intervention [31] and report the data following the Preferred Reporting Items for Systematic Reviews and Meta-Analyses (PRISMA) statement [32] or the Preferred Reporting Items for Systematic Reviews and Meta-Analyses for Children (PRISMA-C) which will be published soon. This protocol follows the Preferred Reporting Items for Systematic Reviews and Meta-Analyses Protocol 2015 (PRISMA-P), which is included as Additional file 1 [33].

### Primary outcome

The patient, intervention, comparator, outcome (PICO) question formulated for this study will be as follows: In children aged less than 18 years of age (P), do the CYP2C9, VKORC1, and CYP4F2 genetic polymorphism (I) compared with the CYP2C9, VKORC1, and CYP4F2 genetic wild type (C) impact on the variability of maintenance dose of patient receiving warfarin (O)?

The detail of the outcome is as follows:

1. Mean difference of warfarin maintenance dose between CYP2C9 variant type (CYP2C9 *1/*2, CYP2C9 *1/*3, CYP2C9 *2/*2, CYP2C9 *2/*3, or CYP2C9 *3/*3) and wild type (CYP2C9 *1/*1) in children
2. Mean difference of warfarin maintenance dose between VKORC1 variant type (VKORC1-1639GA, VKORC-1639AA, VKORC1-1173CT, or VKORC1-1173CT) and VKORC wild type (VKORC1-1639GG or VKORC1-1173TT) in children
3. Mean difference of warfarin maintenance dose between CYP4F2 variant type (CYP4F2-CT or CYP4F2-TT) to that in the CYP4F2 wild type (CYP4F2-CC) in children

We will combine the data regarding haplotypes of VKORC1-1173C>T and VKORC1-1639G>A because these two SNPs have a strong linkage disequilibrium [34]. The unit of mean difference of warfarin maintenance dose will be expressed as milligrams per kilogram per day. When warfarin maintenance dose, which is standardized to milligrams per kilogram per day, is not applicable, we will use standardized mean differences with 95 % confidence interval (CI) for synthesis. Also, we will conduct subgroup analysis according to the literature using milligrams per kilogram per day as the outcome measure.

### Search strategy and sources

A comprehensive literature review search using the OVID platform will be conducted by a specialized librarian, including MEDLINE, EMBASE, Cochrane Central Register of Controlled Trial libraries, as well as gray literature. We will identify relevant studies in any language using medical subject headings (MeSH terms) and text words related to “warfarin” AND “target SNPs” AND “children.” Details of the search strategy are provided in Additional file 2. An age filter (all children, 0 to 18 years) will be used in the MEDLINE and EMBASE plus EMBASE classics search. We will also include studies published only as abstract or conference reports. Additionally, we will hand-search the reference lists of each eligible study included in the present and previous systematic reviews to identify additional eligible articles. In addition, the International Clinical Trials Registry Platform Search Portal and ClinicalTrials.gov will be searched for ongoing or recently completed trials and the international prospective register of systematic reviews (PROSPERO) will be searched for ongoing or recently completed systematic reviews. As relevant studies are identified, reviewers will check for additional relevant cited and citing articles. The principal investigators of studies will be contacted to clarify study eligibility if required. Primary authors will also be contacted to ascertain missing or unpublished data and their knowledge of any other studies not retrieved by our primary search.

Our systematic review protocol was registered with the PROSPERO on 27 February 2015 (registration number: CRD42015016172). If this protocol needs any amendments, we will provide the date of each amendment, describe the change, and give the rationale, accordingly. Changes will not be incorporated into the protocol.

### Eligibility criteria

This review will focus on the potential association between warfarin maintenance dose and genotypes of CYP2C9, VKORC1, or CYP4F2 in children aged less than 18 years old. We will define warfarin maintenance dose as the dose to achieve three consecutive international normalized ratio (INR) measurements within a therapeutic range over a minimum of 4 weeks. We will include original studies which meet the following criteria: (1) observational (cohort or cross-sectional) studies or randomized controlled trials in warfarin-treated pediatric patients; (2) genotypes of CYP2C9, VKORC1, or CYP4F2 were reported; (3) children receiving warfarin for at least 1 month; and (4) maintenance doses of warfarin are presented according to each genotype. There were no restrictions in the inclusion criteria regarding patient demographic information including body weight, height, use of concurrent drugs, indication for warfarin use, and target INR ranges. We will exclude case–control studies, case reports, case series, reviews, editorials, news, and original studies which did not report the outcome of interest in both the children and adults. Duplicated studies will be also excluded. Pharmacogenetic studies involving only adult population aged 18 or older will be excluded. When a study involves both adult and pediatric patients, we will exclude the study if the mean or median age of the study population exceeded 18 years old and subgroup analysis regarding children is unavailable.

### Data collection and analysis

#### Study selection

Two reviewers (MT and TK) will independently screen titles and abstracts of all the retrieved bibliographic records. The inclusion and exclusion criteria will be used for each screening step. If no abstract is available, the full text will be obtained unless the article can be confidently excluded by its title alone. If there is any doubt whether a study should be excluded, the study will proceed to the full-text screen to reduce the likelihood of incorrectly excluding relevant studies. Full text of potentially eligible studies will be independently retrieved by the two reviewers. Disagreements at these screening levels (title/abstract and full text) will be resolved through discussion or adjudication of a third reviewer (SI).

#### Data extraction

After determination of the initial study, eligibility information will be extracted for each study pertaining to study identification (first author, year of publication, and country where patients’ recruitment took place), study design, patient population (number of enrolled patients, age, gender, predominant ethnicity, concomitant drug use, diet, indication for warfarin treatment, target INR range), and primary outcome measurement (allele frequencies and warfarin maintenance dose for each genotype of CYP2C9, VKORC1, or CYP4F2). A list of the eligibility information is presented in Additional file 3. In the case of duplicated studies, we will use the study which had the largest number of patients. We will contact the original authors for missing data if necessary. In order to standardize the unit of warfarin dose to milligrams per kilogram per day, we will also contact the original authors to obtain the data. The two reviewers will independently extract data of all eligible studies and summarize. The disagreement between the reviewers will be resolved by discussion. To ensure consistency across reviewers, we will conduct pre-training of data extraction before starting the review.

#### Data management

Endnote X7 software will be used to upload literature search results and de-duplicated by information regarding author, year of publication, title, and reference type. The Drop Box software, an Internet-based software program that facilitates collaboration among reviewers during the study selection process, will be used to store the literature search results and PDF files of retrieved paper. The uploaded data will be used only for the purposes stated in this study and will not be forwarded to third parties.

#### Assessing a risk of bias of individual studies

Two reviewers will independently assess the risk of bias of all eligible studies in accordance with the Newcastle–Ottawa Scale (NOS) [35]. In addition, the Strengthening the Reporting of Genetic Association study (STREGA) statement will also be utilized [36]. The STREGA statement represents an extension of the Strengthening the Reporting of Observational Studies in Epidemiology (STROBE) [37] and advocates for transparency how a study was done by assessing a 12-item checklist which are important to consider in genetic association studies. These will be undertaken by two separate reviewers. Where there is disagreement, a third reviewer will be used as an arbitrator.

### Data synthesis and presentation

We will weigh the studies by using the inverse variance method and express the effect of each genetic variant on warfarin maintenance doses as mean differences of the doses between the wild type and the respective variant with 95 % CI. When warfarin maintenance dose, which is standardized to milligrams per kilogram per day, is not applicable, we will use the standardized mean differences with 95 % CI for synthesis. When only the median and range are available after contacting primary authors, an imputation method proposed by Hozo et al. [38] will be utilized. Anticipating the heterogeneity between studies, we will combine the data using the DerSimonian and Laird random effect model [39]. A two-sided *P* value less than 0.05 indicates a nominally significant overall association. All statistical analyses will be performed by using the Review Manager version 5.3 (the Nordic Cochrane Centre, Copenhagen, Denmark) or EZR (Saitama Medical Center, Jichi Medical University, Saitama, Japan) [40]. If quantitative synthesis is not appropriate, we will provide a systematic narrative synthesis with information presented in the text and tables to summarize and explain the characteristics and findings of the included studies.

#### Heterogeneity measures

We will assess the magnitude of inconsistency in the study results by the *I*^2^ statistic which expresses the heterogeneity as the percentage of total variation. We will arbitrarily define the three categories of heterogeneity as follows: *I*^2^ <30 %, low; *I*^2^ 30–70 %, moderate; and *I*^2^ >70 %, high. If high levels of heterogeneity among the studies are observed (*I*^2^ ≥ 50 %), the study design and characteristics in the included studies will be analyzed. We will try to explain the source of heterogeneity by subgroup analysis or sensitivity analysis.

#### Publication bias

Publication bias will be assessed using linear regression test of the funnel plot asymmetry. A *P* value less than 0.10 will be considered to have publication bias. If asymmetry is suggested by a visual assessment, we will perform exploratory analyses to investigate and adjust it (trim and fill analysis).

#### Planned sensitivity and subgroup analysis

Subgroup analyses will be used to explore possible sources of heterogeneity, based on the following: (1) patient characteristic (age, predominant ethnicity, indication for warfarin); (2) target INR (median target range <2.5 or ≥2.5); and (3) literature using milligrams per kilogram per day as the outcome measure. Sensitivity analyses will be performed in order to explore the source of heterogeneity as follows: (1) quality components (full-text publication vs. abstracts) and (2) quality of studies by omitting studies that are judged to have lower quality.

#### GRADE framework

The proposed review will use the Grading of Recommendations Assessment, Development and Evaluation (GRADE) guidelines to determine the quality and strength of recommendations [41]. Quality will be adjudicated as high (further research is very unlikely to change our confidence in the estimate of effect), moderate (further research is likely to have an important impact on our confidence in the estimate of effect and may change the estimate), low (further research is very likely to have an important impact on our confidence in the estimate of effect and is likely to change the estimate), or very low (very uncertain about the estimate of effect).

## Discussion

We hope that our systematic review and meta-analysis will appropriately evaluate the effects of CYP2C9, VKORC1, and CYP4F2 on warfarin maintenance dose in children. Even though some previously published studies explored the effect of genotypes on warfarin maintenance dose, the role of genotypes in pediatric patients remains controversial. On the one hand, Biss et al. [23] reported that VKORC1 and CYP2C9 affected the maintenance dose in 120 pediatric patients receiving warfarin, in addition to Shaw et al. [28], who has also reported that warfarin dose variability was attributed to VKORC1 and CYP2C9 polymorphisms in 93 pediatric patients. Similarly, there were some literatures reporting that VKORC1 polymorphism had an effect on warfarin maintenance dose in small sample size [22, 25, 27, 29]. Conversely, Kamal El-Din et al. [30] showed that age was the most significant determinant of the warfarin dose and CYP2C9 and VKORC1 polymorphisms did not have a major impact on the warfarin dose in 41 Egyptian pediatric patients. Nowak-Gottl et al. [22] and Nguyen et al. [27] also reported CYP2C9 had no significant effect on warfarin maintenance dose in 34 pediatric patients and 50 pediatric patients with heart disease, respectively. In this circumstance, Zhang et al. [42] conducted a meta-analysis and have reported that VKORC1-1639 polymorphism had an effect on warfarin dose in pediatric patients in 2015. However, they did not follow the Cochrane Handbook for Systematic Reviews of Intervention or the PRISMA guidelines considered the current gold standard guideline in the systematic review field. Their protocol of meta-analysis was not peer reviewed. In addition, the patients in the two literatures they selected may overlap and they did not include data of some literature meeting the eligibility criteria in the meta-analysis. To avoid these flaws, we will conduct the systematic review and meta-analysis officially following to the principle of the Cochrane Handbook for Systematic Reviews of Intervention and PRISMA guideline. Moreover, we will to utilize the STREGA checklist in addition to NOS to assess the quality of study. This approach will enable us to check whether the eligible literature includes the essential information related to genetic association study.

This study will benefit clinician by providing a summary of effect of polymorphism on warfarin maintenance dose. The results may also have an impact on patient care and facilitate the development of a pharmacogenetic-guided warfarin dosing algorithm in children.

## Abbreviations

CI, confidence interval; CYP2C9, cytochrome P450 2C9; CYP4F2, cytochrome P450 4F2e; GRADE, Grading of Recommendations Assessment, Development and Evaluation; INR, international normalized ratio NOS, the Newcastle–Ottawa Scale; PRISMA, Preferred Reporting Items for Systematic Reviews and Meta-analyses; PRISMA-C, Preferred Reporting Items for Systematic Reviews and Meta-analyses—Reporting for Children; PRISMA-P, Preferred Reporting Items for Systematic Reviews and Meta-analyses Protocol; PROSPERO, international prospective register of systematic reviews; SNPs, single nucleotide polymorphisms; STREGA, Strengthening the Reporting of Genetic Association study; STROBE, Strengthening the Reporting of Observational Studies in Epidemiology; VKORC1, vitamin K epoxide reductase

## Additional files

Additional file 1: PRISMA-P checklist: recommended items to address in a systematic review protocol. (DOC 80 kb) Additional file 2: Search terms and strategies. The search strategy utilized is outlined in more detail in the file. (DOCX 23 kb) Additional file 3: A list of the eligibility information. (PDF 3601 kb)
