# Supplementary material for: Effect of CYP2C9, VKORC1, and CYP4F2 polymorphisms on warfarin maintenance dose in children aged less than 18 years: a protocol for systematic review and meta-analysis
Source: Syst Rev. 2016 Jun 23;5:105. doi: 10.1186/s13643-016-0280-y (PMC4917995; doi:10.1186/s13643-016-0280-y)
Supplement: Additional file 2: — Search terms and strategies. The search strategy utilized is outlined in more detail in the file. (DOCX 23 kb) [file 13643_2016_280_MOESM2_ESM.docx]

**Additional file 2: Search terms and strategies**

1. **Search strategy for MEDLINE**

1 Warfarin/ (15784)

2 ("1 (4' hydroxy 3' coumarinyl) 1 phenyl 3 butanone" or "3 (alpha acetonylbenzyl) 4 hydroxycoumarin" or "3 acetonylbenzonyl 4 hydroxy coumarinedimethylaminoethanol" or "3 alpha phenyl beta acetylethyl 4 hydroxycoumarin" or "3(alpha acetonylbenzyl)4 hydroxycoumarin" or "4-hydroxy-3-(3-oxo-1-phenylbutyl)-2h-1-benzopyran-2-one" or "acetonylbenzylhydroxycoumarin" or "adoisine" or "alpha acetonylbenzyl 4 hydroxycoumarin dimethylaminoethanol" or "antrombin* " or "befarin" or "carfin" or "circuvit" or "compound 42" or "coumadan" or "coumadin" or "coumadine" or "coumafene" or "coumaphene" or "dagonal" or "farin" or "jantoven" or "kumatox" or "maforan" or "marevan" or "orfarin" or "panwarfarin" or "panwarfin" or "prothromadin" or "simarc-2" or "sofarin" or "tedicumar" or "tintorane" or "uniwarfin" or "warfarin*" or "waran" or "warf compound 42" or "warfant" or "warfar" or "warfil 5" or "warfilone" or "warnerin").mp. (23896)

3 1 or 2 (23896)

4 exp Cytochromes/ (105933)

5 Vitamin K Epoxide Reductases/ (869)

6 cytochrome*.mp. (146458)

7 cyp2c9*.mp. (3855)

8 cyp 2c9*.mp. (261)

9 cypiic9*.mp. (0)

10 cyp iic9*.mp. (0)

11 cyp4f2*.mp. (309)

12 cyp 4f2*.mp. (9)

13 menadione epoxide reductase*.mp. (0)

14 menadione oxide reductase*.mp. (0)

15 phylloquinone epoxide reductase*.mp. (1)

16 (vitamin k* adj3 (epoxidase* or reductase* or oxide*)).mp. (1118)

17 Vkorc1*.mp. (843)

18 exp Polymorphism, Genetic/ (215675)

19 polymorphism*.mp. (277641)

20 4 or 5 or 6 or 7 or 8 or 9 or 10 or 11 or 12 or 13 or 14 or 15 or 16 or 17 or 18 or 19 (428730)

21 (infan* or newborn* or new-born* or neonat* or child* or adolescen* or juvenile or teen* or girl* or boy* or youth* or toddler* or paediatric* or pediatric*).mp. (3609048)

22 3 and 20 and 21 (128)

1. **Search strategy for EMBASE plus EMBASE classics search**

1 warfarin/ (72511)

2 ("1 (4' hydroxy 3' coumarinyl) 1 phenyl 3 butanone" or "3 (alpha acetonylbenzyl) 4 hydroxycoumarin" or "3 acetonylbenzonyl 4 hydroxy coumarinedimethylaminoethanol" or "3 alpha phenyl beta acetylethyl 4 hydroxycoumarin" or "3(alpha acetonylbenzyl)4 hydroxycoumarin" or "4-hydroxy-3-(3-oxo-1-phenylbutyl)-2h-1-benzopyran-2-one" or "acetonylbenzylhydroxycoumarin" or "adoisine" or "alpha acetonylbenzyl 4 hydroxycoumarin dimethylaminoethanol" or "antrombin* " or "befarin" or "carfin" or "circuvit" or "compound 42" or "coumadan" or "coumadin" or "coumadine" or "coumafene" or "coumaphene" or "dagonal" or "farin" or "jantoven" or "kumatox" or "maforan" or "marevan" or "orfarin" or "panwarfarin" or "panwarfin" or "prothromadin" or "simarc-2" or "sofarin" or "tedicumar" or "tintorane" or "uniwarfin" or "warfarin*" or "waran" or "warf compound 42" or "warfant" or "warfar" or "warfil 5" or "warfilone" or "warnerin").mp. (75928)

3 1 or 2 (75928)

4 exp cytochrome/ (168870)

5 vitamin K epoxide reductase/ (369)

6 cytochrome*.mp. (192738)

7 cyp2c9*.mp. (5063)

8 cyp 2c9*.mp. (344)

9 cypiic9*.mp. (0)

10 cyp iic9*.mp. (0)

11 cyp4f2*.mp. (497)

12 cyp 4f2*.mp. (10)

13 menadione epoxide reductase*.mp. (33)

14 menadione oxide reductase*.mp. (0)

15 phylloquinone epoxide reductase*.mp. (2)

16 (vitamin k* adj3 (epoxidase* or reductase* or oxide*)).mp. (1107)

17 Vkorc1*.mp. (1470)

18 exp genetic polymorphism/ (339094)

19 polymorphism*.mp. (360053)

20 4 or 5 or 6 or 7 or 8 or 9 or 10 or 11 or 12 or 13 or 14 or 15 or 16 or 17 or 18 or 19 (590997)

21 (infan* or newborn* or new-born* or neonat* or child* or adolescen* or juvenile or teen* or girl* or boy* or youth* or toddler* or paediatric* or pediatric*).mp. (3953862)

22 3 and 20 and 21 (319)

1. **Search strategy for Cochrane Central Register of Controlled Trial**

1 Warfarin/ (1105)

2 ("1 (4' hydroxy 3' coumarinyl) 1 phenyl 3 butanone" or "3 (alpha acetonylbenzyl) 4 hydroxycoumarin" or "3 acetonylbenzonyl 4 hydroxy coumarinedimethylaminoethanol" or "3 alpha phenyl beta acetylethyl 4 hydroxycoumarin" or "3(alpha acetonylbenzyl)4 hydroxycoumarin" or "4-hydroxy-3-(3-oxo-1-phenylbutyl)-2h-1-benzopyran-2-one" or "acetonylbenzylhydroxycoumarin" or "adoisine" or "alpha acetonylbenzyl 4 hydroxycoumarin dimethylaminoethanol" or "antrombin* " or "befarin" or "carfin" or "circuvit" or "compound 42" or "coumadan" or "coumadin" or "coumadine" or "coumafene" or "coumaphene" or "dagonal" or "farin" or "jantoven" or "kumatox" or "maforan" or "marevan" or "orfarin" or "panwarfarin" or "panwarfin" or "prothromadin" or "simarc-2" or "sofarin" or "tedicumar" or "tintorane" or "uniwarfin" or "warfarin*" or "waran" or "warf compound 42" or "warfant" or "warfar" or "warfil 5" or "warfilone" or "warnerin").mp. (2563)

3 1 or 2 (2563)

4 exp Cytochromes/ (1331)

5 Vitamin K Epoxide Reductases/ (29)

6 cytochrome*.mp. (2340)

7 cyp2c9*.mp. (250)

8 cyp 2c9*.mp. (25)

9 cypiic9*.mp. (0)

10 cyp iic9*.mp. (0)

11 cyp4f2*.mp. (13)

12 cyp 4f2*.mp. (0)

13 menadione epoxide reductase*.mp. (0)

14 menadione oxide reductase*.mp. (0)

15 phylloquinone epoxide reductase*.mp. (0)

16 (vitamin k* adj3 (epoxidase* or reductase* or oxide*)).mp. (41)

17 Vkorc1*.mp. (49)

18 exp Polymorphism, Genetic/ (2539)

19 polymorphism*.mp. (4307)

20 4 or 5 or 6 or 7 or 8 or 9 or 10 or 11 or 12 or 13 or 14 or 15 or 16 or 17 or 18 or 19 (6327)

21 (infan* or newborn* or new-born* or neonat* or child* or adolescen* or juvenile or teen* or girl* or boy* or youth* or toddler* or paediatric* or pediatric*).mp. (171892)

22 3 and 20 and 21 (18)

23 Warfarin/ (1105)

24 ("1 (4' hydroxy 3' coumarinyl) 1 phenyl 3 butanone" or "3 (alpha acetonylbenzyl) 4 hydroxycoumarin" or "3 acetonylbenzonyl 4 hydroxy coumarinedimethylaminoethanol" or "3 alpha phenyl beta acetylethyl 4 hydroxycoumarin" or "3(alpha acetonylbenzyl)4 hydroxycoumarin" or "4-hydroxy-3-(3-oxo-1-phenylbutyl)-2h-1-benzopyran-2-one" or "acetonylbenzylhydroxycoumarin" or "adoisine" or "alpha acetonylbenzyl 4 hydroxycoumarin dimethylaminoethanol" or "antrombin* " or "befarin" or "carfin" or "circuvit" or "compound 42" or "coumadan" or "coumadin" or "coumadine" or "coumafene" or "coumaphene" or "dagonal" or "farin" or "jantoven" or "kumatox" or "maforan" or "marevan" or "orfarin" or "panwarfarin" or "panwarfin" or "prothromadin" or "simarc-2" or "sofarin" or "tedicumar" or "tintorane" or "uniwarfin" or "warfarin*" or "waran" or "warf compound 42" or "warfant" or "warfar" or "warfil 5" or "warfilone" or "warnerin").mp. (2563)

25 23 or 24 (2563)

26 exp Cytochromes/ (1331)

27 Vitamin K Epoxide Reductases/ (29)

28 cytochrome*.mp. (2340)

29 cyp2c9*.mp. (250)

30 cyp 2c9*.mp. (25)

31 cypiic9*.mp. (0)

32 cyp iic9*.mp. (0)

33 cyp4f2*.mp. (13)

34 cyp 4f2*.mp. (0)

35 menadione epoxide reductase*.mp. (0)

36 menadione oxide reductase*.mp. (0)

37 phylloquinone epoxide reductase*.mp. (0)

38 (vitamin k* adj3 (epoxidase* or reductase* or oxide*)).mp. (41)

39 Vkorc1*.mp. (49)

40 exp Polymorphism, Genetic/ (2539)

41 polymorphism*.mp. (4307)

42 26 or 27 or 28 or 29 or 30 or 31 or 32 or 33 or 34 or 35 or 36 or 37 or 38 or 39 or 40 or 41 (6327)

43 (infan* or newborn* or new-born* or neonat* or child* or adolescen* or juvenile or teen* or girl* or boy* or youth* or toddler* or paediatric* or pediatric*).mp. (171892)

44 25 and 42 and 43 (18)
