# Supplementary material for: Effect of CYP2C9, VKORC1, and CYP4F2 polymorphisms on warfarin maintenance dose in children aged less than 18 years: a protocol for systematic review and meta-analysis
Source: Syst Rev. 2016 Jun 23;5:105. doi: 10.1186/s13643-016-0280-y (PMC4917995; doi:10.1186/s13643-016-0280-y)
Supplement: Additional file 3: — A list of the eligibility information. (PDF 3601 kb) [file 13643_2016_280_MOESM3_ESM.pdf]

STUDY ELIGIBILITY FORM

FACTORS	ASSESSMENT	COMMENTS
**TYPE OF STUDY**		
1. Is the study described as observational (cohort, case control, or cross-sectional) study or randomized controled trial?	Yes ◉ Unclear ○ No ○<br>**Exclude**	
**PARTICIPANTS**		
2. Were participants treated with warfarin?	Yes ◉ Unclear ○ No ○<br>**Exclude**	
3. Were participants of the postspecified age?<br><br>NB: Please answer „Yes“, If mix age participants i.e. both <=18 years and >18 years are included and state it as comments.<br>No: If only > 18 years.	Yes ◉ Unclear ○ No ○<br>**Exclude**	Subgroups available?
**Genotyping**		
4. Were study participants performed SNPs analysis (CYP2C9, VKORC1, or CYP4F2)?	Yes ◉ Unclear ○ No ○<br>**Exclude**	
**OUTCOMES**		
5. Did the study report warfarin maintenane dose?	Yes ◉ Unclear ○ No ○<br>**Exclude**	
**FINAL DECISION**<br><br>1 X “No” = EXCLUDE<br><br>1 X “Unclear” = UNCLEAR	Include ◉ Unclear ○ Exclude ○	

ORGANISATIONAL ASPECTS					
REF ID		Reviewer, Date		Checked by	
Author, Year					
Journal/Source				Study ID	
Country of origin					
Publication type	Fulltext ☐ / Abstract ☐ / Book chapter ☐ / internal progress report ☐ other (please specify) ☐ brief communication				
Other relevant publications in DE-form	several paper from same DB				
Fate	Decision pending ☐ / Check references ☐ / Use for discussion ☐ EX without listing ☐ / EX with listing ☐ / Other (please specify) ☐				
Notes / Short description					

REASONS FOR EXCLUSION OF STUDY FROM REVIEW (PLEASE SPECIFY according to protocol	
Methods	No original reports ☐ / Other ☐
Patients	No warfarin exposure ☐ age ☐ Subgroups available? ☐
Outcomes	No PGx data avilable ☐ No data for relevant subgroup extractable ☐
Other	Duplicate publication ☐ (ID) / Other:
NONE	Included ☐

CURRENT STATUS
**Question to author**
**Status verified with study investigators or sponsors: Yes** ☐ / **No** ☐ Enter name of the source (e.g. PI, sponsor, etc.)
**Contact address:**

STUDY CHARACTERISTICS	
Sample size	Randomized ☐ / Recruited ☐ / Number ( )
Number of excluded patients	No ☐/ NA ☐/ Yes ☐(N=)
Recruitment method	consecutive inclusion ☐ / N/A ☐ / Others ☐ ( )
Setting	in-patient ☐ / out-patient ☐ / unclear ☐ / NR ☐
Location of trial	
Dates of Recruitment	From ( ) To ( )<br>*Day/Month/Year* *Day/Month/Year*
Study Design	RCT ☐ / Cohort ☐ / Cross-sectional ☐ / Others ☐<br>Single center ☐/ Multicenter trial: international ☐/ national ☐/ # centers:__<br>Prospective ☐ / Retrospective ☐
Aim of study	Warfarin dose requirement ☐ / Time to stabel dose ☐<br>ADE (e.g. severe bleeding or thrombosis) ☐<br>Others ☐ ( )
Funding	Industry ☐/ Public ☐ / Mixed ☐ (industry supported: drug ☐/<br>data management ☐ / travel ☐ / salary ☐ / other ☐<br>unclear ☐ / NR ☐
Conflict of interest statement	Yes ☐ / No ☐ / NR ☐
Tested SNPs	**CYP2C9**: Yes ☐ / No ■<br>*2 (rs1799853) ☐<br>*3 (rs1057910) ☐<br>Others ( )<br>P<0.05?<br>Yes ☐ / No ☐/ NA ■<br><br>**VKORC1**: Yes ☐ / No ■<br>G1639A (rs9923231) ☐<br>C1173T (rs9934438) ☐<br>Others ( )<br>P<0.05?<br>Yes ☐ / No ☐/ NA ■<br><br>**CYP4F2**: Yes ☐ / No ■<br>V433M (rs2108622) ☐<br>Others ( )<br>P<0.05<br>Yes ☐ / No ☐/ NA ■
Definition of warfarin mainteinance dose	
Comments	

PATIENTS CHARACTERISTICS	
Age (year)	Mean ( ) / Median ( ) / Mode ( )<br>SD ( ) / SE ( ) / Minimum ( ) / Maximum ( )<br>1st Quartile (/ 3rd Quartile ( )<br>95% CI inferior ( ) / 95% CI superior ( )
Gender (Number)	Male ( ) / Female ( )
Race (Number)	Caucasian ☐ (88) / Asian ☐ ( )<br>African ☐ ( ) / Others ☐ ( )<br>Comment:
Concomitant drug information	NA ☐ / Available ☐
Diet information	NA ☐ / Available ☐
Indication for warfarin	Congenital or aquired heart disease ☐ (N=)<br>Fontan ☐ (N=) / Heart valve replacement ☐ (N=)<br>Cardiomyopathy ☐ (N=) / Coronary aneurysms ☐ (N=)<br>Pulmonary hypertension ☐ (N=)<br>Other cardiac disorders ☐ (N= ,)<br>Non-Cardiac disorder ☐ (N=)<br>Deep vein thrombosis ☐ (N=) / Stroke ☐ (N=)<br>Other conditions ☐ (N= ,)
Target INR range	NA ☐ / Available ☐
Comments	

OUTCOME MEASUREMENT (Warfarin Mainteinance dose)										
	**Data avilable**	**Sample number**	**Unit**	**Mean**	**Median**	**SD**	**MIN**	**MAX**	**95% CI Inferior**	**95% CI Superior**
CYP2C9										
*1/*1	☐									
*1/*2	☐									
*1/*3	☐									
*2/*3	☐									
*2/*2	☐									
*3/*3	☐									
Variant Hetrozygus	☐									
Variant Homozyguns	☐									
Variant (any *2 or*3)	☐									
VKORC1										
1639-GG	☐									
1639-GA	☐									
1639-AA	☐									
1639-GA or -GG	☐									
1173-CC	☐									
1173-CT	☐									
1173-TT	☐									
1173-CT or -TT	☐									
CYP4F2										
V433M, TT	☐									
V433M, CT	☐									
V433M, CC	☐									
V433M, CT or CC	☐									

Newcastle-Ottawa Quality Assessment Scale for Cohort Studies

Selection

1) Representativeness of the variant genotypes in each SNP
- ☐ a) truly representative in the community ☆
- ☐ b) somewhat representative in the community ☆
- ☐ c) selected group of users
- ☐ d) no description of the derivation of the cohort

2) Selection of the wild genotype in each SNP
- ☐ a) drawn from the same community as the exposed cohort ☆
- ☐ b) drawn from a different source
- ☐ c) no description of the derivation of the non exposed cohort

3) Ascertainment of genotype
- ☐ a) secure record ☆
- ☐ b) structured interview ☆
- ☐ c) written self report
- ☐ d) no description

4) Demonstration of genotyping was not present at start of study
- ☐ a) yes ☆
- ☐ b) no

Comparability

1) Comparability of cohorts on the basis of the design or analysis
- ☐ a) study controls for body size or age ☆
- ☐ b) study controls for any additional factor (e.g. target INR range, indication, or diet) ☆

Outcome

1) Assessment of outcome
- ☐ a) independent blind assessment ☆
- ☐ b) record linkage ☆
- ☐ c) self report
- ☐ d) no description

2) Was follow-up long enough for outcomes to occur
- ☐ a) yes (more than 1 month of stable warfarin control) ☆
- ☐ b) no

3) Adequacy of follow up of cohorts
- ☐ a) complete follow up - all subjects accounted for at least 1 months ☆
- ☐ b) subjects lost to follow up unlikely to introduce bias - small number lost <10 % ☆
- ☐ c) follow up rate < 90% and no description of those lost
- ☐ d) no statement

Total number of stars: stars
